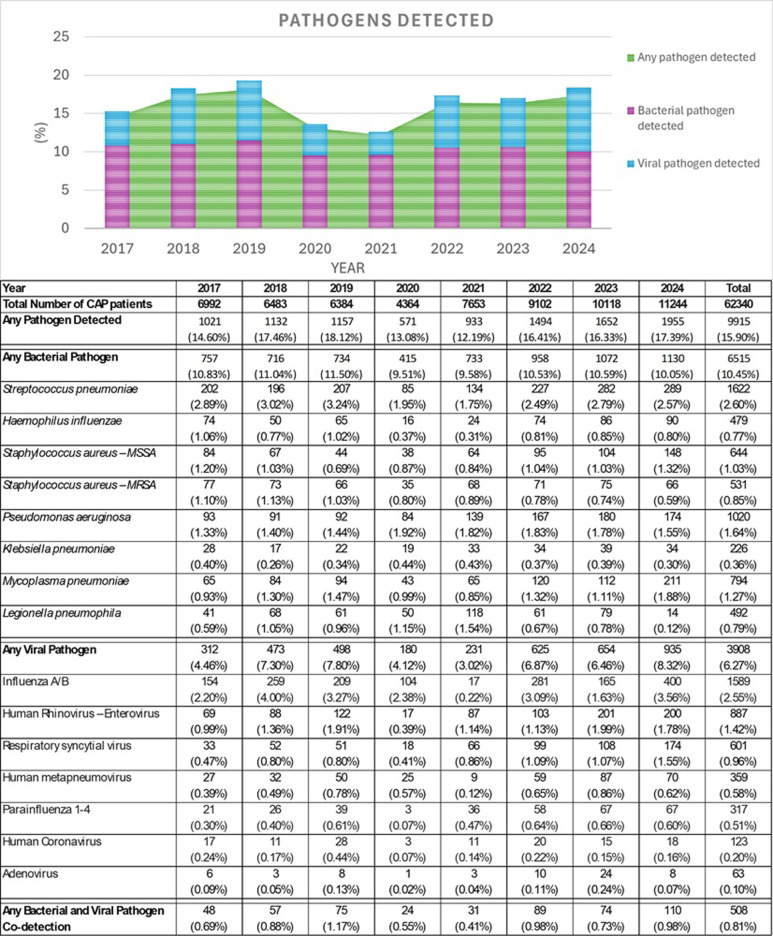# 20 Evaluation of Disinfection Methods and Effects for Handwashing Sinks Contaminated with Pseudomonas aeruginosa

**DOI:** 10.1017/ash.2026.10468

**Published:** 2026-06-23

**Authors:** Nak-Hyun Kim, Danielle Cook, Nora Fino, Claire Ciarkowski, Tejal Gandhi, Lindsay Petty, Scott Flanders, Jennifer Horowitz, David Ratz, Ashwin Gupta, Preeti Misra, Anurag Malani, Valerie Vaughn

**Affiliations:** 1 Seoul National University Bundang Hospital; 2 University of Utah; 3 University of Michigan Medical School; 4 Michigan Medicine; 5 DIvision of Hospital Medicine, Michigan Medicine; 6 University of Michigan; 7 University of Michgian/VA Ann Arbor Healthcare System; 8 Trinity Health Livonia Hospital; 9 Trinity Health Michigan; 10 University of Utah School of Medicine

## Abstract

**Background:** Community-acquired pneumonia (CAP), a heterogeneous clinical entity with a wide range of inciting pathogens, remains a significant cause of morbidity and mortality worldwide. The recent COVID-19 pandemic and subsequent shifts in clinical and diagnostic practices necessitate an updated understanding of the contemporary epidemiologic and etiologic landscape for non-COVID CAP. **Method:** This retrospective cohort study included adult patients hospitalized with CAP between January 1, 2017, and December 31, 2024, across 68 hospitals participating in the Michigan Hospital Medicine Safety consortium. Eligible patients included hospitalized adults with a pneumonia discharge code, symptoms and imaging consistent with pneumonia, and receipt of antibiotic treatment on hospital day 1 or 2. Patients admitted to intensive care, those with concomitant infections, positive SARS-CoV-2 tests, or severe immunosuppression were excluded. Diagnostic testing methods and the pathogens detected were reviewed and summarized by year. **Result:** A total of 62,340 patients were included. Mean age was 69.0 ±15.9 years, and 50.3% (31,359) were male. 94.0% (58,613) received a chest X-ray and 53.1% (33,084) received a CT, with use of CTs increasing over time. Microbiological evaluation was performed in 92.8% (57,820) of patients, with a median 4 [IQR 2-6] etiologic tests per patient. Blood cultures were the most common microbiologic test (74.7%; 46,566) with use slightly decreasing over the study period. In contrast, the use of molecular respiratory pathogen assays increased substantially from 28.2% in 2017 to 77.1% in 2024 (overall performed in 55.6%, 34,655). Over the entire study period, one or more pathogens were detected in 15.9% (9,915) of patients: 10.5% (6,515) had a bacterial pathogen, 6.3% (3,908) had a viral pathogen, and 0.8% (508) had both. The most common pathogens were Streptococcus pneumoniae (2.6%), influenza virus (2.6%), Pseudomonas aeruginosa (1.6%), and human rhinovirus (1.4%). **Conclusion:** The 2019 CAP guideline recommendations to limit traditional bacterial tests to severe CAP patients appear to have had minimal impact on diagnostic practices. Despite a substantial post-pandemic increase in molecular diagnostic use, pathogen detection rates remained low, revealing an etiology in only a minority of patients. These findings underscore the need for a reassessment of the diagnostic approach to optimize resource allocation and ensure high-value testing in hospitalized CAP patients.

**Figure:**
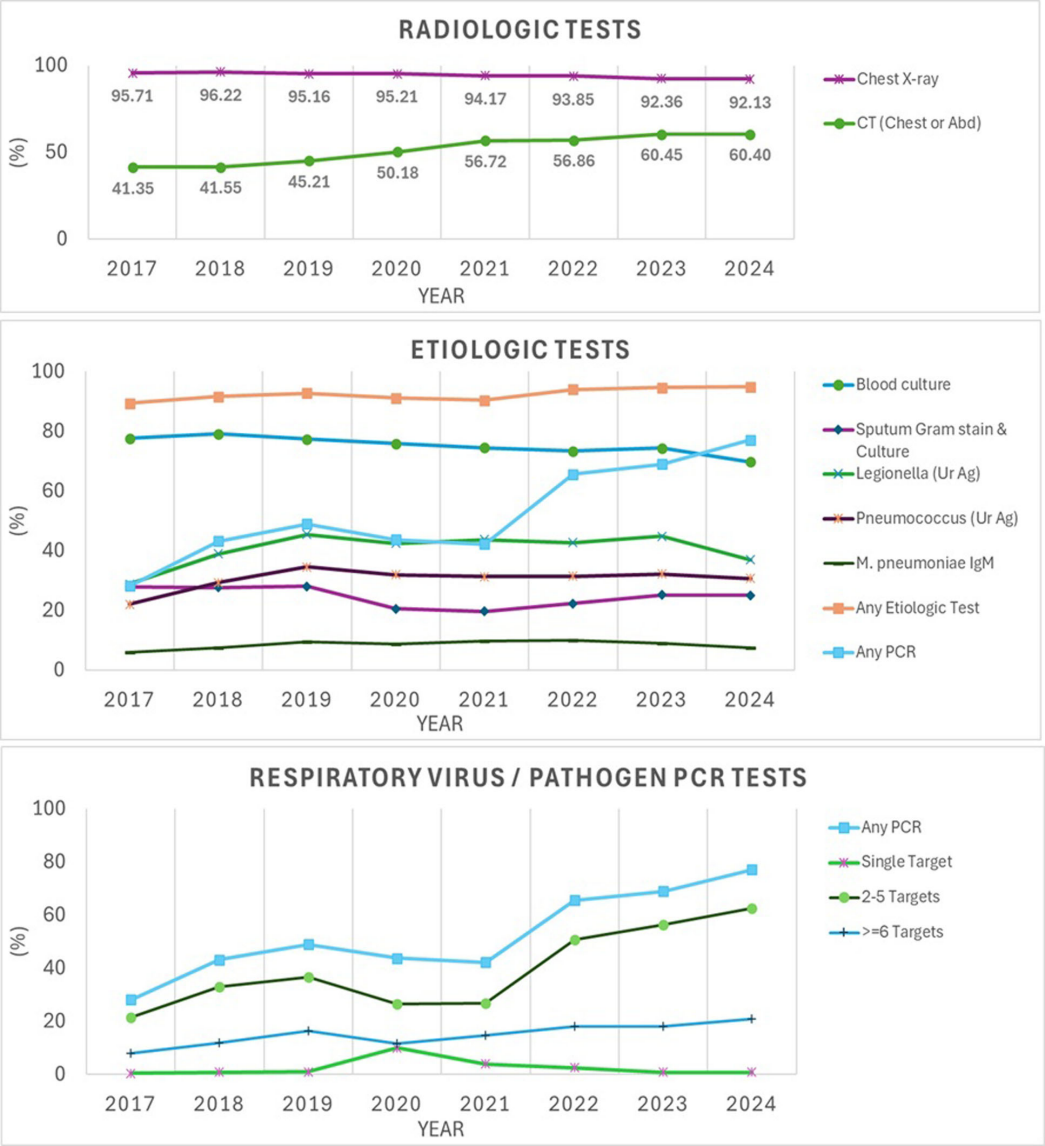

**Figure:**